# Molecular Identification of Two Thioredoxin Genes From *Grapholita molesta* and Their Function in Resistance to Emamectin Benzoate

**DOI:** 10.3389/fphys.2018.01421

**Published:** 2018-10-25

**Authors:** Zhong-Jian Shen, Yan-Jun Liu, Xu-Hui Gao, Xiao-Ming Liu, Song-Dou Zhang, Zhen Li, Qing-Wen Zhang, Xiao-Xia Liu

**Affiliations:** Department of Entomology, MOA Key Laboratory of Pest Monitoring and Green Management, College of Plant Protection, China Agricultural University, Beijing, China

**Keywords:** *Grapholita molesta*, Thioredoxins, RNA interference, oxidative stress, antioxidant defense

## Abstract

Thioredoxins (Trxs), a member of the thioredoxin system, play crucial roles in maintaining intracellular redox homeostasis and protecting organisms against oxidative stress. In this study, we cloned and characterized two genes, *GmTrx2* and *GmTrx-like1*, from *Grapholita molesta*. Sequence analysis showed that *GmTrx2* and *GmTrx-like1* had highly conserved active sites CGPC and CXXC motif, respectively, and shared high sequence identity with selected insect species. The quantitative real-time polymerase chain reaction results revealed that *GmTrx2* was mainly detected at first instar, whereas *GmTrx-like1* was highly concentrated at prepupa day. The transcripts of *GmTrx2* and *GmTrx-like1* were both highly expressed in the head and salivary glands. The expression levels of *GmTrx2* and *GmTrx-like1* were induced by low or high temperature, *E. coli*, *M. anisopliae*, H_2_O_2_, and pesticides (emamectin benzoate). We further detected interference efficiency of *GmTrx2* and *GmTrx-like1* in *G. molesta* larvae and found that peroxidase capacity, hydrogen peroxide content, and ascorbate content all increased after knockdown of *GmTrx2* or *GmTrx-like1*. Furthermore, the hydrogen peroxide concentration was increased by emamectin benzoate and the sensitivity for larvae to emamectin benzoate was improved after *GmTrx2* or *GmTrx-like1* was silenced. Our results indicated that *GmTrx2* and *GmTrx-like1* played vital roles in protecting *G. molesta* against oxidative damage and also provided the theoretical basis for understanding the antioxidant defense mechanisms of the Trx system in insects.

## Introduction

Reactive oxygen species (ROS) is a collective term for hydrogen peroxide, superoxide anion, and hydroxyl radical. It can be generated during metabolic activities and induced by external factors such as pro-oxidants, heavy metals, pesticides, and other adverse factors (Imlay, 2003). Excessive ROS can disrupt the balance of intracellular redox homeostasis (Droge, 2002) and cause oxidative damage to proteins, lipids, and nucleic acids (Wu et al., 2011). For protecting against the toxicity of excessive ROS, aerobic organisms have developed complex antioxidant enzymatic systems to maintain the intracellular ROS at proper levels (Kobayashi-Miura et al., 2007). The thioredoxin (Trx) is an important part of thioredoxin systems that play important roles in redox-regulatory processes (Lu and Holmgren, 2014).

Thioredoxins (Trxs) are the family of soluble proteins involved in cellular dithiol-disulfide redox of organisms (Rietsch and Beckwith, 1998). The Trxs can protect cells from the damage of ROS generated under stressful conditions by acting as disulfide reductases or electron donors in the reduction of disulfide and dithiol (Stryer et al., 1967; Holmgren, 1979; Nakamura et al., 1997; Kalinina et al., 2008; Mahmood et al., 2012). It plays a crucial role in the maintenance of cellular thiol redox balance (Arnér and Holmgren, 2000; Chen et al., 2002; Myers et al., 2008).

In insects, research on Trx has been focused on a few species of insects, for example, *Drosophila melanogaster*, *Bombyx mori*, and *Apis cerana cerana*. In *D. melanogaster*, three Trx genes (Trx1, Trx2, and TrxT) have been identified (Kanzok et al., 2001; Bauer et al., 2002; Svensson et al., 2003), and *DmTrx2* played important roles in redox regulation, the oxidative defense system, and modulating the lifespan of flies (Bauer et al., 2002; Svensson and Larsson, 2007). In *B. mori*, *BmTrx* has been shown to have a major role in resisting oxidative stress caused by extreme temperatures and microbial infection (Kim et al., 2007). In *Apis mellifera*, *AmTrx1* was found in the mitochondria, which is an organelle responsible for aerobic respiration, suggesting *AmTrx1* might be involved in scavenging ROS in mitochondria (Corona and Robinson, 2006). In *A. cerana cerana*, some Trxs, including *AccTrx-like1*, *AccTrx1*, and *AccTrx2* have been demonstrated to participate in antioxidant defense (Lu et al., 2012; Yao et al., 2013, 2014). In Lepidoptera, *SlTrx1* and *SlTrx2* from *Spodoptera litura* and *HaTrx2* from *Helicoverpa armigera* have been identified and demonstrated to participate in antioxidant defense (Kang et al., 2015; Zhang et al., 2015). These studies suggested that Trxs play important roles in maintaining redox homeostasis and resisting adverse circumstances in insects. However, there are no relevant reports in *Grapholita molesta.*

The *G. molesta* (Busck) is a worldwide pest of stone and pome fruits in most temperate fruit-growing regions (Kirk et al., 2013). Nowadays, due to the larvae’s habits of drilling, insecticides used for egg and neonate are the main means to control *G. molesta*. Unfortunately, organophosphate insecticide resistance has occurred in *G. molesta* (Kanga et al., 2003). It is crucial to understand which genes are involved in the defense mechanism of insects. According to previous studies, Trxs play an important role in elucidating the ROS induced by extreme environments. We hypothesize that Trx2 and Trx-like1 can help to resist the insecticides in *G. molesta*. To elucidate the functions of Trx system genes in *G. molesta*, we have identified Trx2 and Trx-like1 and analyzed their spatio-temporal expression patterns. Moreover, the transcript levels of *GmTrx2* and *GmTrx-like1* were also assessed after various types of stress treatments (low or high temperatures, exposure to *Escherichia coli*, *Metarhizium anisopliae*, H_2_O_2_, and emamectin benzoate infection). After *GmTrx2* or *GmTrx-like1* knockdown, we further examined antioxidant enzyme activities and metabolite amounts. Finally, we detected the content of hydrogen peroxide caused by emamectin benzoate and examined the susceptibility of larvae to emamectin benzoate after *GmTrx2* or *GmTrx-like1* silencing.

## Materials and Methods

### Ethics Statement

In this study, the larvae of oriental fruit moth *G. molesta* were originally collected in the Institute of Pomology in Liaoning province, China. No permissions were required for the insect collection, as the orchards are experimental plots belonging to the Institute of Pomology in Liaoning province. The “List of Protected Animals in China” excludes insects.

### Insect Rearing

The *G. molesta* used in this study were reared on fresh apples and an artificial diet at a constant temperature of 25 ± 1°C at 70 ± 10% RH and with a 15 L: 9 D light regime in our laboratory. Adults were reared in beakers (1 L in volume) with fresh apples inside for egg laying and fed with 10% honey solution. The fifth instars larvae picked out from rotten apples were transferred to finger-shaped glass tube (5.5 cm in length × 2.2 cm in diameter) on artificial diet until the pupation.

### Total RNA Extraction and cDNA Synthesis

The TRIzol reagent (TaKaRa, Kyoto, Japan) was used to extract total RNA according to the instructions. The quality and quantity of the extracted RNA products were determined by using an ultraviolet spectrophotometer (Abs260) and a PrimeScript RT Reagent Kit with gDNA Eraser (TaKaRa, Kyoto, Japan); the first-strand complementary DNA (cDNA) was synthesized from 1 μg of total RNA according to the manufacturer’s instructions.

### Primer Design and Quantitative Real-Time Polymerase Chain Reaction Amplification Conditions

The DNAClub and DNAman software were used to design *GmTrx2* and *GmTrx-like1* primers for reverse transcription PCR (RT-PCR), quantitative real-time PCR (qRT-PCR), and dsRNA synthesis. The glyceraldehyde-3-phosphate dehydrogenase (GAPDH, KJ094948.1) and actin (actin, KF022227.1) were used as internal reference genes for the qRT-PCR to normalize target gene expression. The primers were synthesized by Sangon Biotechnology Co., Ltd. (Shanghai, China).

The qRT-PCR was conducted in a 20 μl reaction volume comprising 1 μl cDNA, 10 μl 2 × SYBR Green Supermix (TaKaRa, Kyoto, Japan), 2 μl qRT-PCR primers, and 7 μl ddH_2_O on a Bio-Rad CFX Connect Real-Time PCR Detection System (Hercules, CA, United States). The amplification was performed following the program as: 95°C for 30 s, followed by 40 cycles at 95°C for 5 s, and 60°C for 30 s. The 2^−ΔΔCT^ method was used for the quantitative analysis (Livak and Schmittgen, 2001).

### Cloning, Sequencing, and Sequence Analysis of *GmTrx2* and *GmTrx-like1*

The genes of *GmTrx2* and *GmTrx-like1* were obtained from *G. molesta* transcriptome. The full-length cDNA sequences of both Trx genes were amplified with specific primers (Supplementary Table S1) and *G. molesta* cDNA. According to the manufacturer’s instructions of the DNA fragment purification kit (TaKaRa, Kyoto, Japan) and the pMD^TM^18-T Vector Cloning Kit (TaKaRa, Kyoto, Japan), the PCR products were sequentially purified and cloned into the PMD 18-T vector. The recombinant plasmid extracted was sequenced by Sangon Biotechnology Co., Ltd. (Shanghai, China).

The online bioinformatics ProtParam tool^1^ was used to analyze the physicochemical properties of *GmTrx2* and *GmTrx-like1*. The related homologous protein sequences from various species were obtained from NCBI database and analyzed using DNAman 6.0.3 software. The conserved domains in *GmTrx2* and *GmTrx-like1* were detected using bioinformatics tools available on the NCBI server^2^. The phylogenetic trees based on amino acid sequence were constructed by using the neighbor-joining method with poisson model, uniform rates, and complete deletion in MEGA 5.10 software. To ensure the accuracy of the tree structure, the tree was created with 1000 replicates.

### Developmental Analysis and Tissue Distribution of *GmTrx2* and *GmTrx-like1*

To determine the spatio-temporal expression pattern of *GmTrx2* and *GmTrx-like1*, samples from different developmental stages and different tissues of larvae were collected. The different developmental stages included eggs, first, second, third, fourth, fifth instar, and prepupa; first, third, fifth, and seventh day pupae; and first and third day adults. The stages of pupae and adults were divided into male and female. The different tissues including the head, epidermis, fat body, midgut, Malpighian tubules, and salivary glands were collected from larvae of the fifth instar. All samples were immediately stored at −80°C for total RNA extraction and cDNA synthesis.

### Effect of Different Types of Stress on the Expression of *GmTrx2* and *GmTrx-like1*

To clarify the effect of various stress on *GmTrx2* and *GmTrx-like1* expression, fifth instar larvae were treated by low temperature (15°C) or high temperature (35°C), *E. coli*, *M. anisopliae*, H_2_O_2_, and emamectin benzoate. In the temperature treatments, the larvae were exposed to 15 and 35°C and larvae in 25°C were as a control; For *E. coli*, *M. anisopliae*, and H_2_O_2_ treatments, the larvae were injected with 1 μL of 1.0 × 105 *E. coli* cells, *M. anisopliae* (1.0 cfu/mL × 105 cfu/mL), and hydrogen peroxide (concentration of 100 mM), respectively; control larvae were injected with an equal volume of phosphate buffered saline. The samples from each group were collected at 0, 6, 12, and 24 h after injection. In insecticide treatments, larvae were soaked in emamectin benzoate (active ingredient concentration: 16.42 mg/L), purchased from Hansen Biologic Science Co., Ltd. (Qingdao, China) for 5 s and samples were collected at 2, 4, and 8 h. The larvae soaked in water were used as the controls. There were 13 larvae in each treatment group and 10 larvae in the control group in one replicate and three replicates were used for each treatment. All samples were immediately stored at −80°C for later total RNA extraction.

### Synthesis of dsRNA and Detecting of RNAi Efficiency

The DNA fragment purification kit (TaKaRa Biotechnology, Dalian, China) was used to purify PCR products, which were synthesized with the primers containing a T7 polymerase promoter sequence (Supplementary Table S1) and cDNA of *G. molesta*. The purified PCR products were used as templates and MEGAscript RNAi Kit (Ambion) was used to synthesize the dsRNA of *EGFP*, *GmTrx2*, and *GmTrx-like1*, according to the manufacturer’s instructions. The synthesized dsRNA products were purified by using MEGAclear columns (Ambion) and redissolved with diethyl pyrocarbonate-treated nuclease-free water. The purity and concentration of dsRNA were measured with ultraviolet spectrophotometry and gel electrophoresis. To determine the interference efficiency of *GmTrx2* and *GmTrx-like1*, fifth instar larvae were injected with approximately 3 μg of dsRNA into proleg using capillary microsyringe. The larvae were injected with *dsEGFP* as a control. One replicate of the treatments injected with dsRNA of *GmTrx2* or *GmTrx-like1* contained 13 larvae, while the treatments injected with *dsEGFP* had 10 larvae. Three replicates were used for each treatment. All samples were collected at 24, 48, and 72 h and stored at −80°C for later detection of gene expression.

### Analysis of Enzymatic Activity and Metabolite Content After Knockdown of *GmTrx2* and *GmTrx-like1*

The samples were collected at 48 and 72 h after dsRNA injection of *EGFP*, *GmTrx2*, and *GmTrx-like1*. The BCA Protein Assay Kit (Nanjing Jiancheng Bioengineering Institute, Nanjing, China) was used to extract and quantify total protein. The capacity of superoxide dismutase and peroxidase were assayed by using superoxide dismutase test kit and peroxidase assay kit (Nanjing Jiancheng Bioengineering Institute, Nanjing, China), respectively. The hydrogen peroxide, and ascorbate test kit (Nanjing Jiancheng Bioengineering Institute, Nanjing, China) were used to quantify the contents of hydrogen peroxide and ascorbate, respectively, according to the manufacturer’s protocols.

### Determination of H_2_O_2_ Contents and Survival Assay

Whole body larval samples were collected at 4 and 8 h after soaking in the emamectin benzoate (active ingredient concentration: 16.42 mg/L) for 5 s. The method for detecting hydrogen peroxide concentration was the same as earlier. In the survival assay, after injecting the 3 μg dsRNA of *GmTrx2* or *GmTrx-like1* for 12 h, the larvae were immersed in emamectin benzoate (active ingredient concentration: 1.642 mg/L) for 5 s. Non-injected and injection of *dsEGFP* larvae were as the controls. The number of dead larvae was recorded in each group for 96 h. The tested insects used for the groups of emamectin benzoate, emamectin benzoate + dsEGFP, emamectin benzoate + dsTrx2, and emamectin benzoate + dsTrx-like1 were 75, 73, 69, and 70, respectively.

### Statistical Analyses

The data are presented as means ± standard error (SE) with three independent replicates. Statistically significant differences in gene expression, the results of enzymatic activity, and metabolite contents were evaluated with pair-wise Student’s *t*-test analysis using SPSS 17.0 software and denoted by ^∗^(0.01 < *P* < 0.05) and ^∗∗^(*P* < 0.01). The data for spatio-temporal expression pattern and mortality were analyzed using ANOVA, followed by a Tukey’s HSD multiple comparison test, and the letters were used to indicate significant differences at *P* < 0.05. Survival curves were analyzed by the method of Kaplan-Meier and statistical significance between survival curves was determined using the log-rank test, when *P*-values were <0.05.

## Results

### Sequence Analysis of *GmTrx2* and *GmTrx-like1*

**FIGURE 1:**
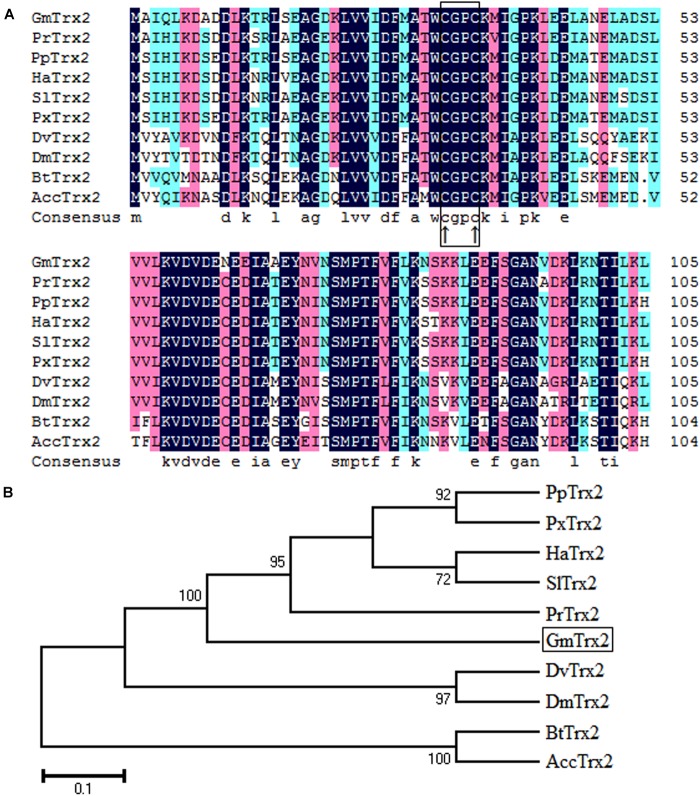
Sequence characterization of Trx2 from various species. **(A)** Multiple alignments of the amino acid sequence of GmTrx2 with homologs from other insect species. Black represents 100% identity, red represents ≥75% identity, green represents ≥50% identity, and white represents <50% identity. The conserved CGPC motif is boxed and the active sites are marked by ↑. *GmTrx2* (*Grapholita molesta*, MH443001), *PrTrx2* (*Pieris rapae*, XP_022125058.1), *PpTrx2* (*Papilio polytes*, NP_001298667.1), *HaTrx2* (*Helicoverpa armigera*, XP_021198961.1), *SlTrx2* (*Spodoptera litura*, XP_022828547.1), *PxTrx2* (*Papilio xuthus*, NP_001299755.1), *DvTrx2* (*Drosophila virilis*, XP_002057775.1), *DmTrx2* (*Drosophila mojavensis*, XP_002002787.1), *BtTrx2* (*Bombus terrestris*, XP_012164907.1), and *AccTrx2* (*Apis cerana cerana*, AFU83101.1). **(B)** Phylogenetic tree analysis of *GmTrx2* and its homologs in insects.

**FIGURE 2:**
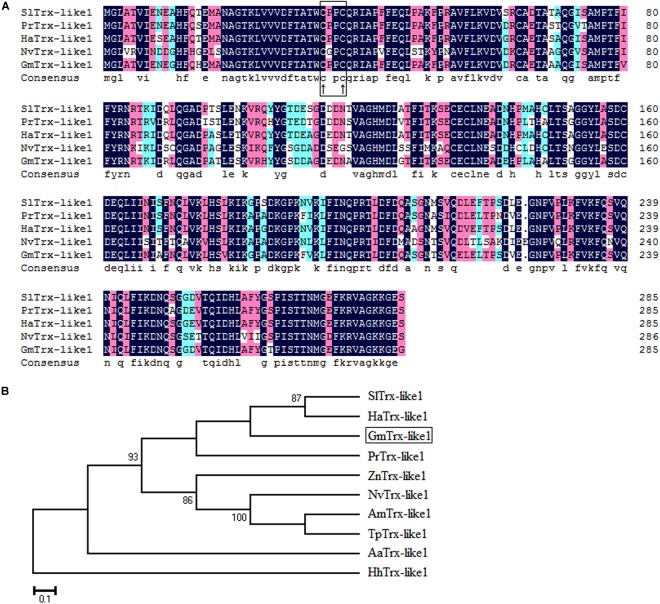
Sequence characterization of Trx-like1 from various species. **(A)** Multiple alignments of the amino acid sequence of *GmTrx-like1* with homologs from other insect species. Black represents 100% identity, red represents ≥75% identity, green represents ≥50% identity, and white represents <50% identity. The conserved CXXC motif is boxed and the active sites are marked by ↑. **(B)** Phylogenetic tree analysis of *GmTrx-like1* and its homologs in insects. *GmTrx-like1* (*Grapholita molesta*, MH443002), *SlTrx-like1* (*Spodoptera litura*, XP_022818589.1), *PrTrx-like1* (*Pieris rapae*, XP_022127554.1), *HaTrx-like1* (*Helicoverpa armigera*, XP_021194378.1), *ZnTrx-like1* (*Zootermopsis nevadensis*, XP_021934497.1), *HhTrx-like1* (*Halyomorpha halys*, XP_014270327.1), *AmTrx-like1* (*Apis mellifera*, XP_623128.1), *NvTrx-like1* (*Nasonia vitripennis*, XP_001608075.1), *AaTrx-like1* (*Aedes aegypti*, XP_001659108.1), and *TpTrx-like1* (*Trichogramma pretiosum*, XP_014235091.1).

Sequence analysis showed the *GmTrx2* gene was 321 bp in length and encoded 106 amino acids with a predicted molecular weight of 11.8 kDa and an isoelectric point of 4.74. Multiple amino acid alignments showed *GmTrx2* had 64–84% identity with homologous sequences of selected species (Figure 1A). The N-terminal portion of the *GmTrx2* had a highly conserved active site sequence of CGPC among all of the selected insect species (Figure 1A). The phylogenetic tree indicated that *GmTrx2* was most closely related to genes from *Pieris rapae* (Figure 1B), which was also consistent with the results of multiple amino acid alignments. The *GmTrx-like1* had an open reading frame of 861 bp and encoded 286 amino acids. The predicted weight of protein was 31.4 kDa and the isoelectric point was 5.48. Multiple alignment analysis of the amino acid sequence showed that *GmTrx-like1* shared amino acid identity (61–88%) with Trx-like1 sequences from selected insect species and the high conserved CXXC motif was found in N-terminal portion (Figure 2A). Phylogenetic analysis showed that *GmTrx-like1* was more closely related to the *SlTrx-like1* and *HaTrx-like1* homolog (Figure 2B).

### Temporal and Spatial Expression Profiles of *GmTrx2* and *GmTrx-like1*

**FIGURE 3:**
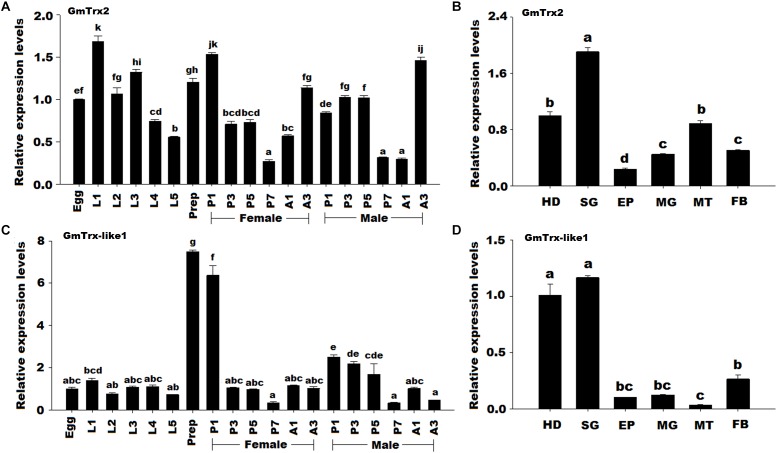
Expression profile of *GmTrx2* and *GmTrx-like1* in different developmental stages and different larval tissues. **(A)** Transcript levels of *GmTrx2* during different developmental stages. Eggs; first, second, third, fourth, fifth instar, and prepupa; first, third, fifth, and seventh day pupae; first, and third day adults. The stages of pupae and adults were divided into male and female. The same as the following. **(B)** Transcript levels of *GmTrx2* in the tissues of fifth instar larvae. HD, heads; EP, epidermis; FB, fat body; MG, midgut; MT, Malpighian tubule; SG, salivary glands; The same as the following. **(C)** Transcript levels of *GmTrx-like1* during different developmental stages. **(D)** Transcript levels of *GmTrx-like1* in the tissues of fifth instar larvae. The data represent the mean ± SE from three biological samples.

The *GmTrx2* was detected in all developmental stages and mainly expressed at stage L1 (first larval day) and stage P1 in female (first day of female pupa) compared to other stages (Figure 3A). For spatial expression, *GmTrx2* had the highest levels of expression in the salivary glands, followed by the head and Malpighian tubules (Figure 3B). The mRNA expression of *GmTrx-like1* was highest at stage prep (prepupa day) and the spatial expression profiles revealed *GmTrx-like1* transcripts were expressed the highest in the head and salivary glands (Figures 3C,D).

### Expression Profiles of *GmTrx2* and *GmTrx-like1* Under Various Oxidative Stresses

**FIGURE 4:**
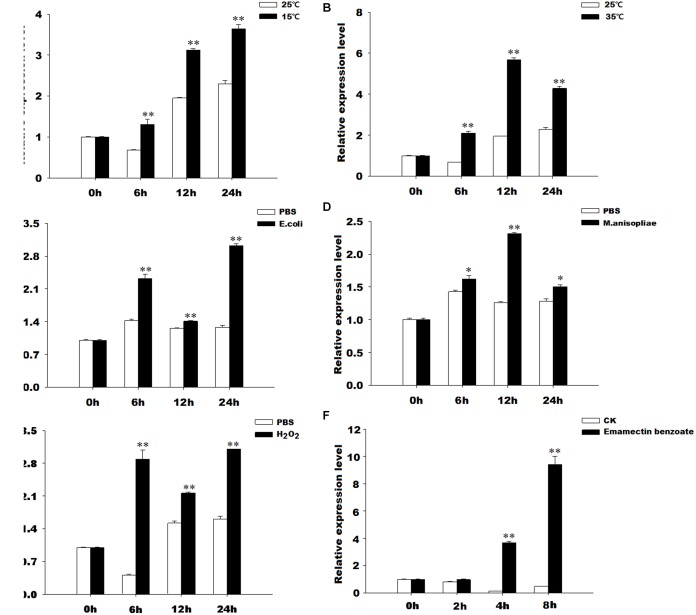
Expression profiles of *GmTrx2* under various stresses. Total RNA was extracted from *G. molesta* samples under different stress challenges, including low temperature (15°C), high temperature (35°C), *E. coli*, *M. anisopliae*, H_2_O_2_, and emamectin benzoate treatments, and then subjected to real-time PCR analysis. **(A–F)** Indicated the expression profiles of *GmTrx-like1* under 15°C, 35°C, *E. coli*, *M. anisopliae*, H_2_O_2_, and emamectin benzoate, respectively. The data represent the mean ± SE of three biological samples. ^∗^0.01 < *P* < 0.05; ^∗∗^*P* < 0.01.

**FIGURE 5:**
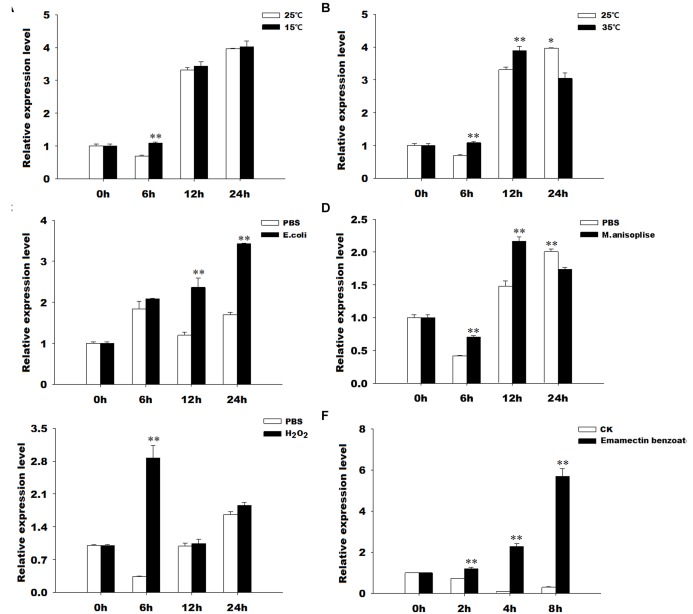
Expression profiles of *GmTrx-like1* under various stresses. Total RNA was extracted from *G. molesta* samples under different stress challenges, including low temperature (15°C), high temperature (35°C), *E. coli*, *M. anisopliae*, H_2_O_2_, and emamectin benzoate treatments, and then subjected to real-time PCR analysis. **(A–F)** Indicated the expression profiles of *GmTrx-like1* under 15°C, 35°C, *E. coli*, *M. anisopliae*, H_2_O_2_, and emamectin benzoate, respectively. The data represent the mean ± SE of 3 biological samples. ^∗^0.01 < *P* < 0.05; ^∗∗^*P* < 0.01.

The results of qRT-PCR revealed that *GmTrx2* was obviously induced by 15 and 35°C, *E. coli*, *M. anisopliae*, and H_2_O_2_ treatments at 6, 12, and 24 h, and then being markedly upregulated at 2, 4, and 8 h after emamectin benzoate immersion (Figure 4). The expression of *GmTrx-like1* was significantly induced at 6 h after 15°C and H_2_O_2_ exposure treatments (Figures 5A,E). At 35°C, *GmTrx-like1* was markedly increased at 6 and 12 h, whereas was suppressed at 24 h (Figure 5B). Under stress of *E. coli*, *GmTrx-like1* transcription was dramatically induced at 12 and 24 h, in addition to being signally enhanced at 6 and 12 h after *M. anisopliae* exposure treatment and obviously induced at 2, 4, and 8 h in response to emamectin benzoate treatment (Figures 5C,D,F).

### Knockdown of *GmTrx2* and *GmTrx-like1* and Effects on Enzymatic Activities and Metabolite Contents After Silencing of the Two Genes

**FIGURE 6:**
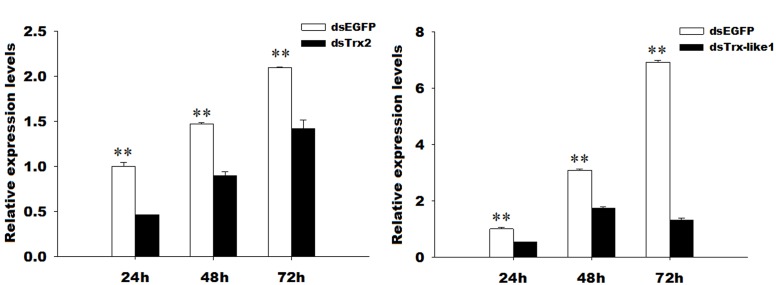
RNA interference efficiency of *GmTrx2* or *GmTrx-like1* dsRNA. **(A)** RNAi-induced reduction of *GmTrx2* expression levels. **(B)** RNAi-induced reduction of *GmTrx-like1* expression levels. The data represent the mean ± SE of three biological samples. ^∗^0.01 < *P* < 0.05; ^∗∗^*P* < 0.01.

The *GmTrx2* and *GmTrx-like1* expressions were significantly inhibited at 24, 48, and 72 h when both the genes were knocked down compared to with EGFP dsRNA injection (Figure 6). The interference efficiency of *GmTrx2* was 53.55, 39.06, and 32.06% at 24, 48, and 72 h (Figure 6A), while *GmTrx-like1* was 46.93, 43.57, and 81% at 24, 48, and 72 h (Figure 6B) after the injection of *GmTrx2* or *GmTrx-like1* dsRNA, respectively.

**FIGURE 7:**
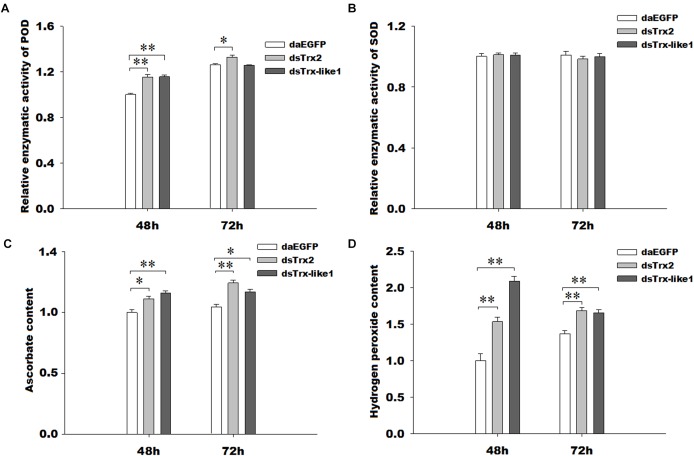
Effects of *GmTrx2* or *GmTrx-like1* knockdown on enzymatic activity of antioxidants **(A)** POD and **(B)** SOD and on the metabolites **(C)** ascorbate and **(D)** hydrogen peroxide. Whole larvae were collected at 48 and 72 h after dsRNA injection of *EGFP*, *GmTrx2*, and *GmTrx-like1* and then analyzed using the corresponding kits according to the manufacturer’s specifications. Each value represents the mean ± SE of three biological samples. ^∗^0.01 < *P* < 0.05; ^∗∗^*P* < 0.01.

The peroxidase capacity after silencing of *GmTrx2* or *GmTrx-like1* was higher than that in the control groups (Figure 7A), while there was no significant change in enzymatic activities of superoxide dismutase (Figure 7B). The contents of hydrogen peroxide and ascorbate were increased at the measured time after knockdown of *GmTrx2* or *GmTrx-like1*, compared with control groups (Figures 7C,D).

### Assay of Oxidant Status *in vivo* Under Emamectin Benzoate and Survival Assay

**FIGURE 8:**
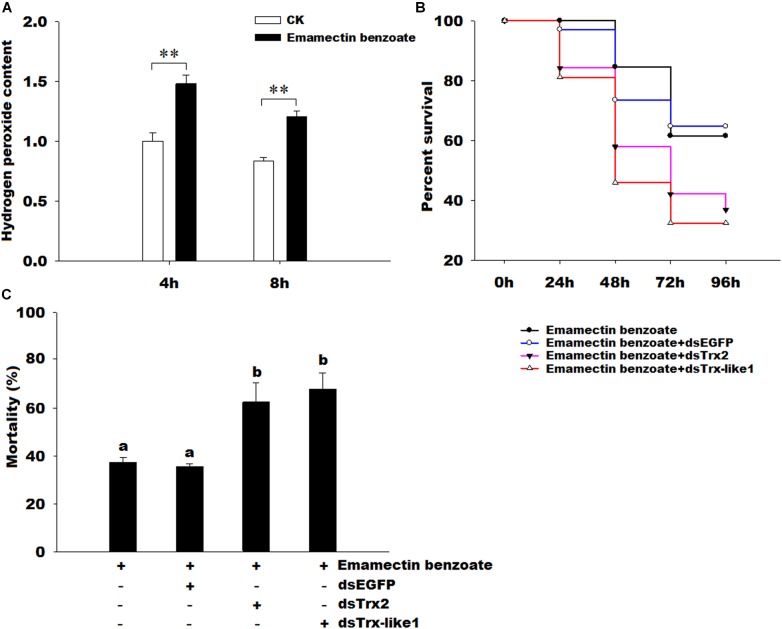
Effects of emamectin benzoate on hydrogen peroxide content and effects of *GmTrx2* or *GmTrx-like1* knockdown on the susceptibility of *G. molesta* larvae to emamectin benzoate. **(A)** Effects of emamectin benzoate on hydrogen peroxide content. The samples of *G. molesta* larvae were collected 4 and 8 h after emamectin benzoate injection and then analyzed using the hydrogen peroxide kits according to the manufacturer’s specifications. **(B)** Survival curves of the larvae soaked in emamectin benzoate after *GmTrx2* or *GmTrx-like1* knockdown; *P* < 0.05. At 12 h after 3 μg dsRNA of *EGFP*, *GmTrx2*, and *GmTrx-like1* infection, the fifth instar larvae were immersed in emamectin benzoate for 5 s. Survival curves were analyzed by the method of Kaplan-Meier and statistical significance between survival curves was determined using the log-rank test, when *P*-values were <0.05. **(C)** The mortality at 96 h after larvae soaked in emamectin benzoate after *GmTrx2* or *GmTrx-like1* knockdown. The data represent the mean ± SE of three biological samples. ^∗^0.01 < *P* < 0.05; ^∗∗^*P* < 0.01. a and b: signification difference, *P* < 0.05.

1To further determine the functions of *GmTrx2* and *GmTrx-like1*, we detected the contents of H_2_O_2_ under emamectin benzoate and the effects of *GmTrx2* or *GmTrx-like1* knockdown on the susceptibility of *G. molesta* larvae exposed to emamectin benzoate. The results showed that H_2_O_2_ concentration was dramatically increased under emamectin benzoate (Figure 8A). Survival curves revealed that *GmTrx2* or *GmTrx-like1* knockdown also obviously promoted susceptibility for *G. molesta* larvae to emamectin benzoate, compared to EGFP dsRNA injection (Figure 8B). The mortality at 96 h of emamectin benzoate (37.26%) and emamectin benzoate + dsEGFP (35.50%) larvae was significantly lower than in emamectin benzoate + dsGmTrx2 (62.49%) or emamectin benzoate + dsGmTrx-like1 (67.94%) larvae (*P* < 0.05, Figure 8C).

## Discussion

Oxidative stress is involved in many different disease processes and causes alterations in the cellular redox state (Jiménez et al., 2006). The Trxs have functions in resisting oxidative damage caused by ROS and maintaining cellular redox homeostasis (Holmgren, 1985; Arnér and Holmgren, 2000; Myers et al., 2008). Previous studies have focused on model insects, for example, *B. mori* (Kim et al., 2007) and *Apis cerana cerana* (Yao et al., 2013). In this study, we focused on the important fruits pest, *G. molesta*. We have identified and characterized *GmTrx2* and *GmTrx-like1* from *G. molesta*. Domain analysis showed that *GmTrx2* and *GmTrx-like1* possessed highly conserved CGPC and CXXC sequences, respectively, suggesting that the two genes belonged to the Trx families.

Temporal expression profiles showed that both *GmTrx2* and *GmTrx-like1* expressions appeared as large fluctuations at the physiological processes of incubation, pupation, and emergence whether male or female. These results suggested that the two genes may play important roles in antioxidant defense in these stages, because these periods of intense physical activity may cause excessive accumulation of ROS. Our transcriptional analysis revealed that the *GmTrx2* and *GmTrx-like1* were expressed at higher levels in the head and salivary glands than other larval tissues. In other insects, *BmTrx* exhibited higher expression in the fat body and silk gland (Kim et al., 2007); *AccTrx1* was mainly expressed in the epidermis (Yao et al., 2014); *HaTrx2* gene was expressed at higher levels in the head and epidermis (Zhang et al., 2015). It revealed that the expression of Trx exhibits a tissue-specific pattern. In addition, the brain tissue was very sensitive to oxidative stress (Rival et al., 2004; Ament et al., 2008) and *AccTrx2* and *AccTrx-like1* had a higher transcript in brain (Lu et al., 2012; Yao et al., 2013), implying that *GmTrx2* and *GmTrx-like1* may play vital functions in the head.

Previous studies have reported that environmental conditions, such as ultraviolet radiation, pesticides, temperature, and heavy metals, can induce oxidative stress (Lushchak, 2011; Kottuparambil et al., 2012). The Trx is a stress-inducible protein and plays significant roles in the scavenging or quenching of oxidants. In *D. melanogaster*, Trx2 has been proved to be closely associated with resistance to oxidative stress (Svensson and Larsson, 2007). The cumene hydroperoxide, indoxacarb, and metaflumizone could stimulate the expression levels of *SlTrx1* and *SlTrx2* in *S. litura* (Kang et al., 2015). The *AccTrx1* and *AccTrx2* from *A. cerana cerana* were upregulated by low or high temperatures, H_2_O_2_, and pesticides (acaricide, paraquat, cyhalothrin, and phoxime) treatments (Yao et al., 2013, 2014). In *H. armigera*, the transcript of *HaTrx2* was significantly stimulated by low or high temperatures, UV light, mechanical injury, microorganism [*E. coli*, *M. anisopliae*, and nucleopolyhedrovirus (NPV)] (Zhang et al., 2015). In this study, *GmTrx2* and *GmTrx-like1* were obviously induced by 15 and 35°C, *E. coli*, *M. anisopliae*, H_2_O_2_, and pesticides-emamectin benzoate treatments, suggesting that *GmTrx2* and *GmTrx-like1* may play critical roles in resisting oxidative stress caused by these adverse conditions.

It was demonstrated that antioxidant enzymes (such as Grx, Trx, and POD) and antioxidant substances [such as ascorbate, protein carbonyl, and glutathione (GSH)] were used to protect organisms by scavenging excess ROS (Meister, 1994; Corona and Robinson, 2006). The changes in antioxidant enzyme activity and metabolite concentrations after gene silencing were also used to demonstrate the function of genes in resisting oxidative stress. For example, after knockdown of *HaGrx*, *HaGrx3*, and *HaGrx5* in *H. armigera* and *AccTrx1* in *A. cerana cerana*, the enzymatic activities of peroxidase, the amount of hydrogen peroxide, and the amount of ascorbate all increased (Yao et al., 2014; Zhang et al., 2016), implying these genes play important roles in resisting oxidative stress. In our study, RNAi mediated knockdown of *GmTrx2*, and *GmTrx-like1* increased the enzymatic activities of POD and the metabolite contents of hydrogen peroxide and ascorbate. It suggested that larvae were exposed to higher oxidative stress after *GmTrx2* and *GmTrx-like1* were silenced, and the genes of *GmTrx2* and *GmTrx-like1* may be involved in protecting *G. molesta* against oxidative stress.

In *A. cerana cerana*, the H_2_O_2_ concentration and the transcripts of *AccTpx5* were induced by phoxim and pyriproxyfen, indicating that oxidative stress might be associated with alterations in *AccTpx5* expression (Yan et al., 2014). In *H. armigera*, the larvae injected by NPV increased ROS production and the lipid damage (Zhang et al., 2015). In this study, emamectin benzoate could dramatically increase the content of hydrogen peroxide and the expressions of *GmTrx2* and *GmTrx-like1*. It revealed that the larvae were exposed to higher oxidative stress and the two genes may be involved in the clearance of hydrogen peroxide caused by insecticide. In order to determine the roles of *GmTrx2* and *GmTrx-like1* involved in protecting organisms from oxidative stress caused by emamectin benzoate, we further examined effects of *GmTrx2* or *GmTrx-like1* interference on the survival curves of emamectin benzoate to *G. molesta* and found that the silencing of the two genes increased the sensitivity of larvae to emamectin benzoate and significantly improved mortality at 96 h, suggesting *GmTrx2* and *GmTrx-like1* were essential in the removal of excessive ROS to protect organisms. At present, the resistance of pests to insecticides was mainly concentrated on important detoxification enzymes such as cytochrome P450. But some antioxidant genes also played an important role in the antagonism of insects, for example, *SlTpx* inhibited *Nomuraea rileyi* infection in *S. litura* (Chen et al., 2014); *HaTrx2* was involved in the defense of larvae against NPV infections in *H. armigera* (Zhang et al., 2015); *GmTrx2* and *GmTrx-like1* protected larvae against emamectin benzoate in our study. Therefore, some antioxidant genes, included *GmTrx2* and *GmTrx-like1*, may become potential targets for insecticide synergists against *G. molesta*.

The Trxs are ubiquitously distributed from Archaea to humans (Lu and Holmgren, 2014). Although it has a conserved domain in all species, Trxs from Archaea to humans have only 27–69% sequence identity to that of *E. coli* Trx1 (Eklund et al., 1991). The sequence inconsistency in a few residues at the amino and carboxyl ends (Eklund et al., 1991) and different mechanisms for reducing Trx (Gleason and Holmgren, 1988) may be responsible for that the physiological functions of Trxs in different types of organisms have evolved from a common fundamental reaction to a large number of different specialized functions. For example, *E. coli* Trx stabilized complexes of bacteriophage T7 DNA polymerase and primed templates (Huber et al., 1987). In bacteria and yeast, Trx served as electron donors of 30-phosphoadenylsulfate (PAPS) reductase (Schwenn et al., 1988; Lillig et al., 1999). In plants, Trx was involved in regulation of chloroplast photosynthetic enzymes (Buchanan, 1991). In mammals, Trx played an important role in the regulation of redox regulation of transcription factors (Schenk et al., 1994), regulation of apoptosis (Saitoh et al., 1998), and immune regulation (Silberstein et al., 1993; Nakamura et al., 1997; Bertini et al., 1999). Therefore, it is very necessary and useful to further study the protein structures, mechanisms, and specific active sites of Trxs in *G. molesta* for finding a suitable way to control pests without affecting other organisms.

## Conclusion

We have characterized two genes, *GmTrx2* and *GmTrx-like1*, and determined their temporal-spatial expression profiles. The expression levels of *GmTrx2* and *GmTrx-like1* were induced by low or high temperature, *E. coli*, *M. anisopliae*, H_2_O_2_, and pesticides such as emamectin benzoate. After knockdown of *GmTrx2* or *GmTrx-like1*, the enzymatic activities of POD and the metabolite contents of hydrogen peroxide and ascorbate all increased. These results revealed that *GmTrx2* and *GmTrx-like1* may play important roles in resistance to excessive ROS. Emamectin benzoate increased the H_2_O_2_ concentration and *GmTrx2* or *GmTrx-like1* were silenced, which improved the sensitivity of larvae to insecticide-emamectin benzoate, further indicating that *GmTrx2* and *GmTrx-like1* play vital roles in protecting *G. molesta* against oxidative damage. These findings may be useful for understanding the antioxidant defense mechanisms of the Trx system in insects.

## Author Contributions

All authors listed have made a substantial, direct, and intellectual contribution to the work. Z-JS and X-XL conceived and designed the experiments. Z-JS and Y-JL performed the experiments. Z-JS, X-ML, S-DZ, and Y-JL analyzed the data. Z-JS, X-ML, S-DZ, and X-HG contributed to reagents, materials, and analysis tools. Z-JS, ZL, Q-WZ, and X-XL wrote the paper.

## Conflict of Interest Statement

The authors declare that the research was conducted in the absence of any commercial or financial relationships that could be construed as a potential conflict of interest.

## Footnotes

1 http://web.expasy.org/protparam/

2 https://www.ncbi.nlm.nih.gov/Structure/cdd/wrpsb.cgi
