# Supplementary material for: Molecular Identification of Two Thioredoxin Genes From Grapholita molesta and Their Function in Resistance to Emamectin Benzoate
Source: Front Physiol. 2018 Oct 25;9:1421. doi: 10.3389/fphys.2018.01421 (PMC6210739; doi:10.3389/fphys.2018.01421)
Supplement: Supplementary file 1 [file Table_1.DOC]

**Table S1.** **Primers used in the current study**.

| **Primer name (Abbreviation)** | **Sequence (5'-3')** | **Tm values (**°C**)** | **Product length (bp)** | **Description** |
| --- | --- | --- | --- | --- |
| GmTrx2-F | GGCCATCCACATCAAAGAC | 50 | 344 | Amplification of open reading frame, forward |
| GmTrx2-R | GAGTACACAGTCCGTTGGC |  |  | Amplification of open reading frame, reverse |
| GmTrx-like1-F | CATTCCCTGATTGTTGTCG | 50 | 959 | Amplification of open reading frame, forward |
| GmTrx-like1-R | GAGGTGCCACTGAGTATC |  |  | Amplification of open reading frame, reverse |
| GmTrx2-qF | GTGGTGATCGACTTCATGG | 60°C for two-step method | 157 | Real-time PCR primer, forward |
| GmTrx2-qR | TGGAGTTGACGTTGTATTCC |  |  | Real-time PCR primer, reverse |
| GmTrx-like1-qF | GTCTAGCCACAGTAATTGAG | 60°C for two-step method | 177 | Real-time PCR primer, forward |
| GmTrx-like1-qR | CGTCGACCTTAAGGAACA |  |  | Real-time PCR primer, reverse |
| GmActin-qF | CTTTCACCACCACCGCTG | 60°C for two-step method | 222 | Real-time PCR primer, forward |
| GmActin-qR | CGCAAGATTCCATACCCA |  |  | Real-time PCR primer, reverse |
| GmGAPDH-qF | GGAAAGCTGACTGGTATGG | 60°C for two-step method | 167 | Real-time PCR primer, forward |
| GmGAPDH-qR | ACCTGGTCCTCGGTGTAG |  |  | Real-time PCR primer, reverse |
| EGFP-RNAiF1 | CCTGAAGTTCATCTGCACCAC | 52 | 538 | The first step PCR primer of dsRNA synthesis, forward |
| EGFP-RNAiR1 | CTCCAGCAGGACCATGTGATC |  |  | The first step PCR primer of dsRNA synthesis, reverse |
| EGFP-RNAiF2 | TAATACGACTCACTATAGGGAGA  CCTGAAGTTCATCTGCACCAC | 60 | 584 | The second step PCR primer of dsRNA synthesis, forward |
| EGFP-RNAiR2 | TAATACGACTCACTATAGGGAGA  CTCCAGCAGGACCATGTGATC |  |  | The second step PCR primer of dsRNA synthesis, reverse |
| GmTrx2-RNAiF1 | AGATGATCGGGCCCAAAC | 52 | 212 | The first step PCR primer of dsRNA synthesis, forward |
| GmTrx2-RNAiR1 | CTTCAGCTTCAGAATGGTG |  |  | The first step PCR primer of dsRNA synthesis, reverse |
| GmTrx2-RNAiF2 | TAATACGACTCACTATAGGGAGA  AGATGATCGGGCCCAAAC | 60 | 258 | The second step PCR primer of dsRNA synthesis, forward |
| GmTrx2-RNAiR2 | TAATACGACTCACTATAGGGAGA  CTTCAGCTTCAGAATGGTG |  |  | The second step PCR primer of dsRNA synthesis, reverse |
| GmTrx-like1-RNAiF1 | ACAGAACAAAGATAGACCG | 52 | 457 | The first step PCR primer of dsRNA synthesis, forward |
| GmTrx-like1-RNAiR1 | TGGAACTTGACGAACTTG |  |  | The first step PCR primer of dsRNA synthesis, reverse |
| GmTrx-like1-RNAiF2 | TAATACGACTCACTATAGGGAGA  ACAGAACAAAGATAGACCG | 60 | 503 | The second step PCR primer of dsRNA synthesis, forward |
| GmTrx-like1-RNAiR2 | TAATACGACTCACTATAGGGAGA  TGGAACTTGACGAACTTG |  |  | The second step PCR primer of dsRNA synthesis, reverse |
